# Analysis of distribution, capacity and utilization of public health facilities in Borno, North-Eastern Nigeria

**DOI:** 10.11604/pamj.2020.35.39.17828

**Published:** 2020-02-12

**Authors:** Usman Alhaji Aliyu, Mustapha Adam Kolo, Muhammad Chutiyami

**Affiliations:** 1 Department of Geography, University of Maiduguri, Borno State, Nigeria; 2 Shehu Sule College of Nursing and Midwifery, Damaturu, Yobe State, Nigeria

**Keywords:** Health facilities, distribution, capacity, socio-economic status, access, utilization, Nigeria

## Abstract

**Introduction:**

This study aimed to analyze the spatial distribution and capacities of public health facilities and assess utilization of the facilities in Biu area of Borno State, Nigeria.

**Methods:**

A descriptive survey of health facilities and households were conducted by stratifying the area into 11 electoral wards. Data collection instruments include a hand-held GPS (Garmin 76CSx) and 2 sets of structured questionnaires (facility and household). The hand-held GPS was used in taking the coordinates of each health facility in the area. Twenty-five facility-based and 400 household-based questionnaires were administered.

**Results:**

It was identified that 138 public health personnel serve the area’s population of 240,838. Medical professionals (doctors/nurses/midwives) to patient ratio is 1:2973, about 7 times less than the minimum WHO recommendation of 2.5 medical personal per 1000 population. Uneven distribution of facilities exists, which impact on utilisation. For instance, a ward (Mandaragrau) with a population of 18,732 have 5 facilities (4 dispensaries and 1 primary health care) in comparison to a ward (Miringa) with a population of 21,343 with only one Dispensary. Income level and distance were significant socio-economic factors affecting service utilisation (p < 0.001). Area’s households Gini index was 26.7, most of which (49.7%) survive on less than USD2/day and majority (33.6%) spend an average cost of treatment of ₦2,750 (approx. $8) per clinic visit.

**Conclusion:**

It was concluded that insufficiency and inequity in distribution of healthcare services exist in Borno State. It is thus recommended that future policies be directed toward improving healthcare in under-served areas.

## Introduction

Healthcare is central to community well-being as well as a fundamental aspect of life. Lack of basic health facilities and services in any community is significantly associated with poor productivity, reduced life expectancy and increased mortality rates [1-5]. This therefore necessitate the need for equity in distribution of health facilities. Both accessibility and utilization are important aspects of equitable distribution of health resources, which is based on needs of the population rather than equal distribution. Health System as an organizational set-up is charged with the responsibility of distributing and servicing the health care needs of a given population [3, 6], thereby achieving positive health outcomes. In developed nations, a tangible proportion of its wealth is budgeted to healthcare provision and sustainability, thus there is a better health outcome. In most developing countries on the other hand, there is need for increased expenditure on healthcare provision and parameters need to be put in place to ensure its sustainability. Awoyemi *et al.* [4] opined that, improvement in healthcare leads to improvement in life expectancy, which serves as a robust indicator of human development.

Evidences have also shown that among the least developed countries, increase in life expectancy is strongly correlated with increase in income/productivity [2, 7-9]. Therefore, there is need for adequate and equitable distribution of healthcare services in any given country, particularly in sub-Saharan Africa, where health outcome is poorest. Healthcare provision in Nigeria is the responsibility of the three tiers of government; the Local, State and the Federal Governments, which handles the primary, secondary and tertiary health facilities respectively. The Federal Government’s role is majorly limited to coordinating the affairs of university teaching hospitals and federal medical centers (tertiary healthcare) while the State Government manages the various general/specialists hospitals (secondary healthcare). The local government on the other hand focus on Primary Health Care (PHC), which is regulated by the Federal Government, through the National Primary Health Care Development Authority (NPHCDA) [10]. Utilization of health services among the Nigerian population is directly associated with accessibility and several socioeconomic variables [9, 11, 12].

This is particularly seen in rural areas of Nigeria and many sub-Saharan African countries, where barriers, notably distance of health facilities impedes utilization [9, 13]. On these notes, Inyang [14] opined that access to health facilities is a function of the degree of fairness in spatial distribution of the facilities. Similarly, Ujoh and Kwaghsende [15] added that the quality of services rendered is directly proportional to the level of manpower available. This explains the need for adequate/qualified health professionals, who can deliver quality services to the people concerned. Studies in this field in Nigeria are limited, most of which use secondary data or Geographic Information System (GIS) to compute distribution/utilization of health facilities [5, 16]. Furthermore, most of these studies were conducted in the Nigerian regions of North-Central [15], South-South [11], South-East [16] or South-West [5,12], North-Western and North-Eastern regions have the poorest health outcomes in the country, with northeast being the worst affected area, partly due to the recent insurgency that characterized the area. Similarly, it is a common norm in developing countries like Nigeria, where most of developmental policies are targeted toward urban areas [17], which could be associated with lack of political-will and/or limited available evidence from rural areas. This study therefore aims to assess the spatial distribution and capacities of public health facilities as well as utilization of the facilities in a semi-urban area of Borno State, North-East Nigeria.

## Methods

**Study design:** this study adopted a descriptive survey of health facilities and households. The study was conducted in Biu Local Government Area (LGA) of Borno State, Nigeria.

**Population and sampling:** the estimated population of Biu L.G.A is 240,838 [18], which serve as the population for this study. The study area was stratified into its eleven electoral wards; Buratai, Miringa, Galtimare, Gunda, Dugja, Sulumtha, Mandaragrau, Yawi, Garubula, Gur and Zarawuyaku. Sample size was deduced using the Yamane’s formula [19] below:

$$\text{n}=\frac{\text{N}}{1+\text{N}{\left(\text{e}\right)}^{2}}$$

Where: n = sample size, N= Total population (240,838), e = significance of error (0.05). Therefore, n = 400 (sample size).

Similarly, Yamane’s formula [19] for determining number of respondents (Sample) was used to determine the sampled population for each ward.

$$\frac{\text{n}400}{\text{N}}$$

Where N = Total population (240,838), n = sub-population size (population of each ward). Hence, 400 copies of questionnaires were administered across the 11 wards of the area (Table 1). A systematic sampling technique was employed by selecting one household after every 5 households in a ward, until the total number of participants is reached in every ward.

**Table 1:** Sampling frame

S/No	Wards in Biu	Population sizes	Sample
1	Buratai	17239	29
2	Miringa	21343	35
3	Galdimare	19021	32
4	Gunda	27217	45
5	Dugja	31317	52
6	Sulumthla	25749	43
7	Mandaragirau	18732	31
8	Yawi	19923	33
9	Garubula	16509	27
10	Gur	15750	26
11	Zarawuyaku	28028	47
	**Total**	240,838	400

**Data collection:** data was collected using a hand-held Global Positioning System (GPS) and two sets of questionnaires (facility and household). The GPS (Garmin 76CSx) was used to obtain and record the geographic locations of the health facilities in the area. The GPS instrument was validated by three independent experts in the field of GIS and remote sensing from University of Maiduguri, Nigeria. It was further checked for reliability by taking several readings of some coordinates, which gives similar readings. At each health facility, the GPS was used to take the geo-location (coordinate) of the health facility. The reading from the GPS was taken after three repeated readings to avoid calibration error from the recording. The facility-based questionnaire was used to obtain information about the facility´s capacity in terms of number of health personnel and bed space. The facility-based questionnaire was administered to each health facility and was filled-in by the head/record officer of each health facility. The household questionnaire was used to assess utilization of health facilities by the population of the area. Head of each household was chosen to fill the questionnaire. The participants´ questionnaire was pilot tested among 30 respondents and modified accordingly, before it was administered to the sampled population. Literate respondents fill the questionnaires themselves, while those that are not English-literates were assisted by research assistants, all of which were health personnel from the surveyed facilities.

**Data analysis:** data was analysed using descriptive statistics with SPSS version 25. The GPS data was analysed with the help of Arc-GIS version 9.3 and presented on a map. Analysis of household survey was based on 386 respondents, who fully completed the questionnaires. This gives a response rate of 96.5%.

## Results

**Figure 1:**
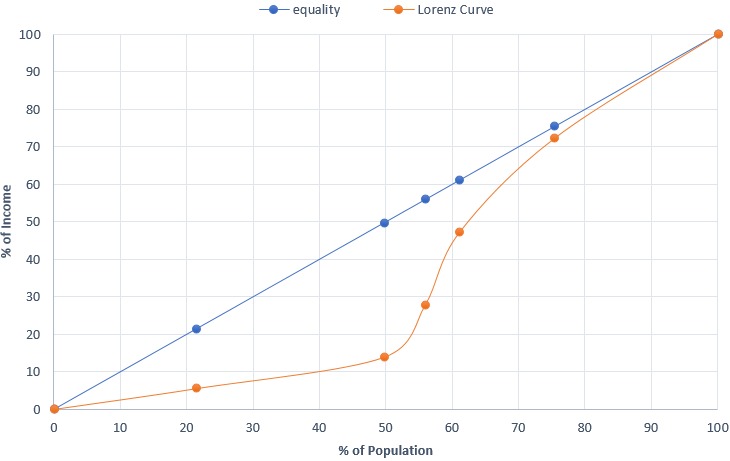
A Lorenz Curve indicating income distribution in Biu LGA, 2016

**Socio-demographic characteristics of respondents:** majority of the respondents that participated in the study are male (72.5%) between the ages of 20 to 30 years (43.5%). This indicates that Biu LGA is largely inhabited by youths in comparison to those over 40 years of age (13.6%). Most participants are married (64%) with secondary and diploma education accounting for 29.8 % and 25.9 % respectively. This shows that majority of Biu population have acquired basic education, although about one-quarter (23.6%) have no formal education. About half are self-employed and have monthly income less than ₦20,000 (USD1 approx. ₦350). In other words, most of the respondents in the study area, including those with family, live on less than $2/day, of which more than 2/5 of those with the low monthly income (<₦20,000) survive on less than $1 per day (Table 2). A Lorenz curve for the population indicated a smaller income difference in the top 50^th^ percentile of the population, however, the lower half of the population shares less than 15% of the income (Figure 1). Accordingly, the estimated Gini index of the population was 26.7, about two-times less than that of the country (48.8), based on the CIA (Central Intelligence Agency) world factbook estimate of 2013. This indicates more equality in wealth distribution in Biu LGA compared to Nigeria as a country.

**Table 2:** Socio-demographic characteristics of respondents (N=386)

Variable	Frequency	Percentage %
**Age**		
≤20	84	22
21-25	90	23.3
26-30	78	20.2
31-35	46	11.9
36-40	35	9.0
41-45	21	5.4
46-50	16	4.1
51-55	12	3.1
>55	4	1.0
**SEX**		
Male	280	72.5
Female	106	27.5
**MARITAL STATUS**		
Married	249	64
Single	100	26
Divorced	10	3
Widow	27	7
**LEVEL OF EDUCATION**		
Non-formal	91	23.6
Primary	40	10.4
Secondary	115	29.8
Diploma	100	25.9
Bachelor degree	35	9
Master/PhD	5	1.3
**OCCUPATION**		
Self employed	212	55
Civil servant	114	30
Unemployed	60	15
**MONTHLY INCOME**		
<₦10,000	83	21.5
₦10,000-19,999	109	28.2
₦20,000-29,999	24	6.2
₦30,000-39,999	20	5.2
₦40,000-49,999	55	14.2
₦50,000 and above	95	24.7

**Utilization of health facility:** majority of the respondents (54.7%) prefer to attend government-owned (public) health facilities, in comparison to chemist (25.4%), traditional/self-medication (9.3%) and private facilities (10.6%). Preference to public facilities was largely due to low cost of treatment (39.8%), despite long distance (61.4%), long waiting time (83.7%), poor attitude of health personnel (63.3%) and poor services (74.1%). Preference to public health facilities was significantly associated with level of income (χ^2^ = 97.3, p < 0.001), whereby majority of respondents with low economic status (0 to ₦19,999) prefer such facilities in comparison to middle-high income earners (Table 3). This indicates the extent to which socioeconomic status impact on utilization of healthcare in Biu. Similarly, a significant association exists between utilization of the health facilities and distance travelled to reach a facility (χ2 = 55.4, p < 0.001). It was identified that majority of the respondents that prefer public health facilities traveled 10km or less to reach a facility, as compared to those travelling above 10km (Table 3). This indicates how distance of health facilities affect access and utilization of the facilities. Furthermore, less than half (48%) of the respondents can transport themselves to health facilities with a vehicle (car/bus/motorcycle), compared to those using other means of transport (Table 3). However, there is no significant relationship between use of the facilities and mode of transportation (χ2 = 8.1, p = 0.148). Common sickness affecting participants in the study area, which requires them to attend a health facility, include malaria (50.33%), diarrhea (23.3%) or typhoid fever (19.4%), all of which are preventable diseases. Accordingly, most of the respondents (33.6 %) claimed to be spending an average cost of treatment of ₦2,750 per visit, while 23.3% spend as low as ₦500 and 9.3% spend as high as ₦5000 or above. Although, based on these figures, about half (50.3%) are likely to afford the basic cost of treatment as they have a monthly average income of at least ₦20000 (above country´s minimum wage). However, those with monthly income of less than ₦20000 (49.7%), otherwise known as those below the 50th percentile on the Lorenz curve (Figure 1) are likely to find it difficult to cope with the average cost of treatment. Moreover, these costs of treatment are mostly found in government-owned facilities and it exclude specialist services like surgery, dental care, scanning or x-rays as revealed by some respondents.

**Table 3:** Pearson chi-square test for preference to public health facilities by income level, distance travelled and mode of transportation

χ2 = 97.3, df5, (p<0.001)			Income Level						
			<₦10,000	₦10,000-19,999	₦20,000-29,999	₦30,000-39,999	₦40,000-49,999	₦50,000 +	Total
Preference to Public Health Facilities	Yes	Count	40	92	19	0	11	49	211
		Expected Count	45.4	59.6	13.1	10.9	30.1	51.9	211.0
		% of Total	10.4%	23.8%	4.9%	0.0%	2.8%	12.7%	54.7%
	No	Count	43	17	5	20	44	46	175
		Expected Count	37.6	49.4	10.9	9.1	24.9	43.1	175.0
		% of Total	11.1%	4.4%	1.3%	5.2%	11.4%	11.9%	45.3%
Total		Count	83	109	24	20	55	95	386
		Expected Count	83.0	109.0	24.0	20.0	55.0	95.0	386.0
		% of Total	21.5%	28.2%	6.2%	5.2%	14.2%	24.6%	100.0%
χ2 = 55.4, df5, (p<0.001)			Distance Travelled						Total
			None	1-5km	6-10km	11-15km	16-20km	21km +	
Preference to Public Health Facilities	Yes	Count	16	4	131	16	35	9	211
		Expected Count	19.7	10.9	98.4	32.8	38.3	10.9	211.0
		% of Total	4.1%	1.0%	33.9%	4.1%	9.1%	2.3%	54.7%
	No	Count	20	16	49	44	35	11	175
		Expected Count	16.3	9.1	81.6	27.2	31.7	9.1	175.0
		% of Total	5.2%	4.1%	12.7%	11.4%	9.1%	2.8%	45.3%
Total		Count	36	20	180	60	70	20	386
		Expected Count	36.0	20.0	180.0	60.0	70.0	20.0	386.0
		% of Total	9.3%	5.2%	46.6%	15.5%	18.1%	5.2%	100.0%
χ2 = 8.1, df5, (p=0.148)			Mode of Transport						Total
			Car	Motorcycle	Bicycle	Canoe	Beast	Walk/Foot	
Preference to Public Health Facilities	Yes	Count	37	67	57	9	15	26	211
		Expected Count	33.9	67.2	62.3	5.5	18.0	24.1	211.0
		% of Total	9.6%	17.4%	14.8%	2.3%	3.9%	6.7%	54.7%
	No	Count	25	56	57	1	18	18	175
		Expected Count	28.1	55.8	51.7	4.5	15.0	19.9	175.0
		% of Total	6.5%	14.5%	14.8%	0.3%	4.7%	4.7%	45.3%
Total		Count	62	123	114	10	33	44	386
		Expected Count	62.0	123.0	114.0	10.0	33.0	44.0	386.0
		% of Total	16.1%	31.9%	29.5%	2.6%	8.5%	11.4%	100.0%

**Figure 2:**
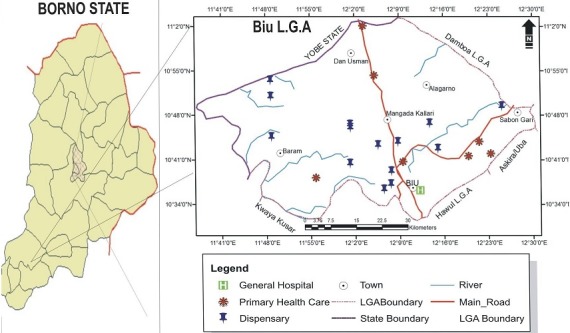
Spatial distribution of health facilities in Biu LGA, 2016

**Distribution of facilities:** secondary data collected from the State ministry of health indicated a total of 25 health facilities in Biu LGA, of which there is one General Hospital (GH), 3 Primary Health Care (PHC), 2 Maternity Centers (MC), 1 Health post (HP) and 18 Dispensaries (D). These facilities were distributed unevenly in the study area (Table 4). For instance: Mandaragrau ward has as many as 5 facilities (4D and 1PHC) while Miringa ward has only one Dispensary, despite the later having a bigger population (21,343) than the former (18,732). Similarly, Dugja ward with the highest population (31,317) has equal number of health facilities (2) as Buratai ward, with about half its population (17,239). The spatial distribution of the facilities as shown on Figure 2 indicated that most of the primary health facilities were concentrated around the LGA headquarters (Sulumthla, Biu) despite the presence of a secondary facility. This left other areas like Gunda ward underserved, which has only one primary health care facility and is farthest from the referral center in Sulumthla ward (Figure 2).

**Table 4:** Distribution of health facilities and personnel by wards in Biu LGA, 2016

S/No	Name of Ward	Population	Facilities	GH	PHC	MC	D	HP	MPs	OHPs
1.	Buratai	17239	2	0	0	0	1	1	0	2
2.	Miringa	21343	1	0	0	0	1	0	0	2
3.	Galdimare	19021	2	0	0	0	2	0	0	4
4.	Gunda	27217	1	0	0	0	1	0	0	2
5.	Dugja	31317	2	0	0	1	1	0	3	4
6.	Mandaragirau	18732	5	0	1	0	4	0	6	5
7.	Yawi	19923	2	0	1	0	1	0	6	6
8.	Garubula	16509	3	0	1	0	2	0	6	3
9.	Gur	15750	2	0	0	0	2	0	0	2
10.	Zarawuyaku	28028	2	0	0	1	1	0	4	4
11.	*Sulumthla	25749	3	1	0	0	2	0	56	23
	**Total**	204,838	25	1	3	2	18	1	81	57

GH=General Hospital, PHC=Primary Health Care, MC=Maternity Centre, D=Dispensary, HP=Health Post, MP=Medical Professionals (doctors, nurses & midwives), OP= Other Health Professionals (Lab Technicians, Community Health Officers, Community Health Extension Workers, Medical Record Officers).

* Sulumthla – Only ward with secondary health facility and all the 6 doctors. Source: Field work, 2016

**Facility capacity:** the public health facilities capacity in-terms of health personnel revealed a total of 138 health professionals. These include 6(4.3%) physicians/doctors, 75(54.3%) nurses/midwives, 8(5.8%) pharmacists, 5(3.6%) laboratory technicians, 37(26.8) community health extension workers (CHEW), 5(3.6%) community health officers (CHO) and 2(1.4%) medical record officers, who are distributed over the eleven (11) wards (Table 4). However, a wide variation exists, with more than half (69%) of these personnel working in the secondary health facility, leading to under staffing in the primary health facilities, where majority of the population attend. The ratio of all the health professionals (138) to the population (240,838) is 1:1745, while for only medical professionals (only doctors/nurses/midwives), the ratio increases to 1:2973. On the other hand, the facilities capacity in-terms of bed spaces, revealed a similar pattern, with more than half of the bed spaces (59.5%) available in the secondary health facility. Unlike the man-power distribution, whereby at least one health personal exists in all the primary health facilities, bed space for at least 24-hour observation/admission were not available in some wards (Dugja and Garubula dispensaries). This left the facilities with the option of referral to other facilities when there is need for admission. Similarly, a ward like Miringa with a population of 21,343 has only 7 bed spaces for admission in its only health facility, while Gunda ward with a population of 27,217 has only 5 bed spaces in its only facility. Other bed spaces distribution in the wards include: Gur (4), Galdimare (5), Buratai (5), Dugja (6), Zarawuyak (13),Yawi (17), Gurumbula (18), Mandaragrau (36) and Sulumthla, with the highest number of beds (220) due to presence of a general hospital.

## Discussion

The study explored how public health facilities were distributed and utilized in a densely populated area in North-Eastern Nigeria. A major strength of this study is its focus on a major semi-urban population with its surrounding communities, as opposed to majority of studies that focus on urban/capital cities. Similarly, it is likely to shape future State´s healthcare policies and planning, to focus more on equity between different communities within an area, rather than just equality approach. The finding of the study indicates that youths form most of the population (43.5%) and the population are educated at least to secondary level (56%), which enables them to be self-employed rather than relying on government employment. Although about half of these population largely depend on small scale businesses and farming but earn a monthly income above the national minimum wage of ₦18,000 ($51). However, the other half survive on less than the minimum wage, sharing less than 15% of the wealth (Figure 1), out of which more than two-fifth were below the poverty line i.e. live on less than $1 per day. People in the latter category are likely to find it difficult to cater for their basic life expenses, let alone covering healthcare expenses.

This is in line with other studies, which put majority of the Nigerian population as living below the poverty line, leading to poor health outcomes [20-22]. The finding of this study further shows majority of the respondents´ preference to government (public) health facilities due to affordability. Preference to government health facilities were also reported in other parts of Nigeria despite providing less quality services, which many literatures associated it with less cost of treatment [5, 9, 11, 23]. Distance travelled to access a health facility is a major determining factor in utilizing care according to the finding of this study. While some respondents travel within a 5km radius to access health services, others need to travel 10km or more to access health services. The uneven distribution of health services is common in many Nigerian regions, particularly in rural areas, where health outcomes are generally poor [4]. Delay in reaching a facility complicates the matter as more than half of respondents (51.2%) were unable to transport themselves to a health facility with a reliable vehicle (car/motorcycle) coupled with lack of functional ambulance services, which could lead to high incidence of preventable deaths in emergency situations.

Delay in reaching a health facility is one of the major delays that affect healthcare in Nigeria [5, 24] and many sub-Saharan African countries [25], most of which complicate maternal and child health outcomes. Distribution of health facilities from the findings of this study shows an uneven distribution and below the recommendation of the NPHCDA [26]. The NPHCDA recommend that a ward in an LGA must have at least one Primary Health Care (PHC) center while a village in a ward with a population of 2000-5000, must be provided with a health clinic (Health Post-HP, Maternity Center-MC or Dispensary-D). However, none of the primary health facilities (PHC, MC, HP and D) meet the standard. The findings indicate 3PHCs, 2MCs, 1HP and 18Ds, which according to the standard [26] should respectively be 11PHC, 11MC, 55HP and 45D. To further complicates things, the distribution tends to be uneven i.e. do not coincide with the population size (Table 4). On the other hand, one secondary health facility exists in the study area, which is in line with the minimum requirement for an LGA in Nigeria, to serve as a referral center for primary health facilities in the LGA [26].

Looking at the facilities´ capacity in-terms of manpower, the finding of this study revealed that medical personnel (doctors/nurses/midwives) were concentrated in the secondary health facility (69%), leaving the primary health facilities staffed with less skilled personnel. Ujoh and Kwaghsende [15] opined that quality of services rendered is directly affected by manpower availability. This indicates that even if there is equity in distribution of health facilities, lack of skilled personnel could lead to poor delivery of the services. The WHO 2006 Global workforce and the 2016 estimate for achieving Sustainable Development Goals (SDGs), respectively indicated a minimum of 2.5 and 4.45 medical staff (physician/nurse/midwife) per 1,000 populations, which is required to provide adequate coverage of primary healthcare [27]. Comparing this standard with the population of this study (240,838), it can be inferred that at least 602 medical staff are required to provide adequate primary care services in Biu LGA of Borno State. However, only 81 medical staff exist in the study area, which is less than 1/7 of the WHO minimum recommendation. A major limitation of this study is its focus on government owned health facilities, which neglect impact of private facilities, hence ratio of facility distribution and patient-professional ratio are likely to be underestimated. However, the proportion of the population that utilize private health facilities were only about one-tenth, indicating a minor difference in-terms of facility distribution or patient-professional ratio.

## Conclusion

Based on the findings of this study, it was concluded that health facilities in the study area were unevenly distributed and the number of primary care facilities and capacities were far below the recommendation of the NPHCDA and WHO. Utilization were more towards government-owned facilities, mainly due to affordability, despite high medical professional-patient ratio (1:2973). However, access to the facilities particularly in the case of emergencies are likely to be poor due to long distance of referral center for wards with limited primary healthcare facilities (notably Gunda and Miringa), coupled with poor road network and lack of operational ambulance services. It is thus recommended that the State government in collaboration with the NPHCDA, be committed toward improving healthcare in under-served areas thereby preventing avoidable mortalities/morbidities and to ensure achievement of SDGs. Further studies in this area could look at health outcomes between different sub-populations with respect to availability of health facilities and/or income level.

### What is known about this topic

- Spatial distribution and utilization of health facilities is well documented in many southern Nigerian States, particularly in the urban areas.

### What this study adds

- Unfold a story in a major semi-urban area with its rural communities in Northern Nigeria;
- Impact of socio-economic status on utilization of health services in an area characterized by conflict for a decade;
- The need for health policies to focus not just on equal distribution of health resources, but equity in the distribution.

## References

[1] Olujimi JAB (2007). Accessibility of rural dwellers to health facilities in Nigeria, the Oyo Region experience. Pakistan Journal of Social Science.

[2] Omrani-Khoo H, Lotfi F, Safari H, James ZB, Moghri J, Shafii M (2013). Equity in distribution of health care resources; assessment of need and access, using three practical indicators. Iranian J Publ Health.

[3] Jin J, Wang J, Ma X, Wang Y, Li R (2015). Equality of medical health resource allocation in China based on the Gini Coefficient Method. Iran J Public Health.

[4] Awoyemi TT, Obayelu AO, Opaluwa HI (2017). Effect of distance on utilization of healthcare services in Rural Kogi State, Nigeria. Journal of Human Ecology.

[5] Ajala AO, Sanni L, Adeyinka SA (2005). Accessibility to healthcare facilities, a panacea for sustainable rural development in Osun State, South-west Nigeria. J HUM Ecol.

[6] Asuzu MC (2004). The necessity for a health systems reform in Nigeria. Journal of community medicine and primary health care.

[7] Deaton A (2003). Health, inequality and economic development. Journal of economic Literature.

[8] Manzoor I, Hashimi NR, Mukhtar F (2009). Determinant and pattern of healthcare services utilization in post graduate students. J Ayuba med coll Abbottabad.

[9] Onah H, Ikeako L, Iloabachie G (2006). Factors associated with the use of maternity services in Enugu, Southeastern Nigeria. Social Science and Medicine.

[10] Nigeria NPHCDA About the National Primary Healthcare Development Authority 2018.

[11] Atser J, Akpan PA (2009). Spatial distribution and accessibility of health facilities in Akwa Ibom State, Nigeria. Ethiopian Journal of Environmental Studies and Management.

[12] Adetunji MA (2013). Spatial distribution, pattern and accessibility of urban population to health facilities in Southwestern Nigeria: the case study of Ilesa. Mediterranean Journal of Social Sciences.

[13] Bour D (2003). Analyzing the primacy of distance in the utilization of health services in the Ahafo-Ano south district, Ghana. The international journal of Health planning and Management.

[14] Inyang IB (1994). Provision of Health Care Facilities in Nigeria: The Problem of Equity and Accessibility. Ibom Journal of Social Issues.

[15] Ujoh F, Kwaghsende F (2014). Analysis of spatial distribution of health facilities in Benue State. Nigeria Scientific and Academic Publishing.

[16] Ifeanyi R, Johnbusco CO, Chijioke GE (2012). Accessibility analysis of healthcare delivery system within Enugu urban area, using Geographic information system. Journal of Geographic information system.

[17] John LS (2005). Planning Sustainable Urban Growth in Nigeria: Challenges and Strategies.

[18] Nigeria National Population Commission NPC (2016). Nigeria Estimated Population.

[19] Yamane T (1967). Statistics, an introduction analysis. Harper and Rao.

[20] Ucha C (2010). Poverty in Nigeria: some dimensions and contributing factors. Global Majority E-Journal.

[21] Garcia RM, Kohl R, Ruengsorn A, Zislin J (2006). Nigeria: Economic Performance Assessment (Washington, DC: United States Agency for International Development (USAID)).

[22] Oluwatayo IB (2009). Explaining inequality and welfare status of households in rural Nigeria: evidence from Ekiti State. Academic Journal of Plant Sciences.

[23] Osubor KM, Fatusi AO, Chiwuzie JC (2006). Maternal health-seeking behavior and associated factors in a rural Nigerian community. Matern Child Health J.

[24] Erim DO, Kolapo UM, Resch SC (2012). A rapid assessment of the availability and use of obstetric care in Nigerian healthcare facilities. PLoS ONE.

[25] Nour NM (2008). An introduction to maternal mortality. Reviews in Obstetrics and Gynecology.

[26] (2018). Nigeria NPHCDA. Publications.

[27] WHO (2016). Health workforce requirements for universal health coverage and the sustainable development goals. Human Resources for Health Observer Series.

